# Still the Joker in the Pack: When to Take Out Cyclosporine in the Game?

**DOI:** 10.1093/ibd/izab066

**Published:** 2021-03-20

**Authors:** Tamás Resál, Kata Szántó, Mariann Rutka, Klaudia Farkas, Tamás Molnár

**Affiliations:** First Department of Medicine, University of Szeged, Faculty of Medicine, Szeged, Hungary

To the Editors,

We have read with interest the recently published article by Dwadasi et al examining the 1-year colectomy-free survival with the use of calcineurin-inhibitor therapy in severe ulcerative colitis (UC) patients.^1^ We agree that it is essential to determine where the right place of an old-school drug is to avoid colectomy during a severe flare-up of UC, considering the novel targeted agents, as well. As most of the data on cyclosporine use in UC comes from the prebiological era, it is necessary to collect as much data as possible to set up an optimal therapeutic order of the current medicines against a severe flare-up. Here we present our experiences on the efficacy and safety of cyclosporine therapy, stratified by prior biologic therapy.

In our institution, intravenous cyclosporine is used at a dose of 4 mg/bwkg as an induction therapy in severe, steroid-refractory UC for 5 to 7 days followed by oral administration. We retrospectively analysed the short- and long-term data of our hospitalized cyclosporine-treated UC patients, focusing on the previous biological therapy.

During the same examined period (from January 1, 2013, to December 31, 2020), 27 inpatients received intravenous cyclosporine at our department. Seventy percent of them also received methyl-prednisolone as a rescue therapy, and 75% had pancolitis. Among the patients, 77.8% had prior exposure to anti-TNF and 7.4% to vedolizumab. During the hospitalization, mean partial Mayo score (pMayo) decreased from 7.6 to 2.3 (*P* < 0.001) and mean C-reactive protein (CRP) level decreased from 66.8 to 15.2 ug/mL (*P* < 0.001). Reduction of CRP levels and pMayo values did not differ significantly between the group naïve to biological treatment or the group exposed to it.

One-year colectomy rate was only 11.1% in our cohort. It should be highlighted that 68% of the patients still received oral cyclosporine at week 52. Duration of cyclosporine use apparently did not have any impact on the rate of colectomy (*P* = 0.8879). Previous biological treatment(s) did not increase colectomy rate (*P* = 0.2) in our cohort. Side effect occurred in 1 case as a mild, reversible elevation of serum urea-nitrogen. Prior biologic treatment did not have impact on the efficacy of induction with cyclosporine and did not increase the rate of 1-year colectomy rate. Our data did not support the limited efficacy of third-line cyclosporine; however, we presented a relatively small number of biologic-naïve and third-line patients. Hence, we agree with the authors that further clinical observations are mandatory to determine the optimal sequence of treatment for taking advantage of maximum efficacy and safety.

## Notes

Supported by: This work was supported by the research grants of the National Research, Development and Innovation Office (Grant ID: 125377, 129266 and 134863), by the New National Excellence Program of the Ministry of Human Capacities (UNKP-20-5-SZTE-161 to FK) and Janos Bolyai Research Grant (BO/00598/19/5) and the Géza Hetényi Research Grant (to FK, RM and BA) by the Faculty of Medicine, University of Szeged.

Author Contributions: All authors reviewed and approved the final version of the manuscript.
